# PS1/*γ*-Secretase-Mediated Cadherin Cleavage Induces *β*-Catenin Nuclear Translocation and Osteogenic Differentiation of Human Bone Marrow Stromal Cells

**DOI:** 10.1155/2016/3865315

**Published:** 2016-12-08

**Authors:** Danielle C. Bonfim, Rhayra B. Dias, Anneliese Fortuna-Costa, Leonardo Chicaybam, Daiana V. Lopes, Hélio S. Dutra, Radovan Borojevic, Martin Bonamino, Claudia Mermelstein, Maria Isabel D. Rossi

**Affiliations:** ^1^Institute of Biomedical Sciences, Federal University of Rio de Janeiro, Rio de Janeiro, RJ, Brazil; ^2^Clementino Fraga Filho University Hospital, Federal University of Rio de Janeiro, Rio de Janeiro, RJ, Brazil; ^3^Institute of Medical Biochemistry, National Institute of Cancer, Rio de Janeiro, RJ, Brazil; ^4^Molecular Carcinogenesis Program, National Institute of Cancer, Rio de Janeiro, RJ, Brazil; ^5^Evandro Chagas Clinical Research Institute, Oswaldo Cruz Institute (FIOCRUZ), Rio de Janeiro, RJ, Brazil

## Abstract

Bone marrow stromal cells (BMSCs) are considered a promising tool for bone bioengineering. However, the mechanisms controlling osteoblastic commitment are still unclear. Osteogenic differentiation of BMSCs requires the activation of *β*-catenin signaling, classically known to be regulated by the canonical Wnt pathway. However, BMSCs treatment with canonical Wnts* in vitro* does not always result in osteogenic differentiation and evidence indicates that a more complex signaling pathway, involving cadherins, would be required to induce *β*-catenin signaling in these cells. Here we showed that Wnt3a alone did not induce TCF activation in BMSCs, maintaining the cells at a proliferative state. On the other hand, we verified that, upon BMSCs osteoinduction with dexamethasone, cadherins were cleaved by the PS1/*γ*-secretase complex at the plasma membrane, and this event was associated with an enhanced *β*-catenin translocation to the nucleus and signaling. When PS1/*γ*-secretase activity was inhibited, the osteogenic process was impaired. Altogether, we provide evidence that PS1/*γ*-secretase-mediated cadherin cleavage has as an important role in controlling *β*-catenin signaling during the onset of BMSCs osteogenic differentiation, as part of a complex signaling pathway responsible for cell fate decision. A comprehensive map of these pathways might contribute to the development of strategies to improve bone repair.

## 1. Introduction

Human bone marrow stromal cells (BMSCs) constitute a heterogeneous population of clonogenic progenitors [1], characterized* in vitro* by the expression of CD90, CD73, CD105, CD146, and the ability to differentiate into osteoblasts, chondrocytes, and adipocytes [2–4]. Due to their proliferative capacity and differentiation potential, BMSCs are envisioned as a tool for bone bioengineering [5, 6]. However, the mechanisms that direct differentiation towards osteoblasts are still not fully understood.

Developmental studies using mice models showed that the differentiation of mesenchymal progenitors into the osteoblastic lineage requires the upregulation of Runx-2 [7, 8] downstream of *β*-catenin signaling [9, 10]. This pathway is classically known to be activated by receptor-mediated canonical Wnt signaling, which turns off the *β*-catenin destruction complex composed by GSK3*β* (Glycogen synthase kinase), Axin, and APC (Adenomatous Polyposis Coli) [11, 12]. Under these circumstances, *β*-catenin translocates to the nucleus, where it forms a complex with TCF/LEF (T Cell Factor/Lymphoid Enhancer Factor) transcription factors to activate gene transcription [11–14].

Nevertheless, attempts to osteoinduce BMSCs with canonical Wnt proteins have shown contradictory results. While some studies showed enhanced osteogenic differentiation [15, 16], others reported increased cell proliferation and impaired differentiation [17, 18]. A possible explanation to these findings came from the observation that the Wnt coreceptor LRP5/6 (low-density lipoprotein receptor-related protein) is frequently associated with the adhesion protein N-cadherin in osteoprogenitor cells, which prevents its activation and the transduction of Wnt signaling [19]. In this way, a more complex transduction signaling pathway, involving the regulation of cadherins, would be required to induce *β*-catenin signaling in these cells [20]. Indeed, sustained N-cadherin expression in osteoprogenitors has been associated to maintenance of the “undifferentiated” state [21–25], and its downmodulation was observed during the progression of osteogenic differentiation* in vitro* and* in vivo* [21, 23, 26–28]. However, how cadherin modulation allows progression towards the osteogenic differentiation pathway is still under scrutiny.

One of the mechanisms that control cadherin stability in the plasma membrane is the proteolytic cleavage mediated by matrix metalloproteases (MMP) and Presenilin-1 (PS1)/*γ*-secretase, an enzymatic complex involved in the proteolysis of several transmembrane proteins, such as Notch [29–34]. Following the cleavage of the amino-terminal domain by a MMP, the membrane-associated, C-terminal fragment (CTF-1) of the cadherin molecule is subsequently cleaved by PS1/*γ*-secretase, generating a second fragment (CTF-2) that is released in the cytosol [30, 34–37]. In vascular smooth cells and embryonic fibroblasts, cadherin cleavage resulted in the release of *β*-catenin from cadherin complexes, followed by its nuclear translocation, which altered cellular functions such as proliferation [38] and migration [39].

Here we evaluated whether cadherin cleavage would occur during BMSCs osteoinduction, as a mechanism regulating *β*-catenin signaling function. We also evaluated the effects of isolated Wnt3a treatment in *β*-catenin-mediated signaling and BMSCs behavior. A comprehensive map of the net of signaling pathways controlling BMSCs osteogenic differentiation will be a fundamental step for the development of strategies for bone repair.

## 2. Materials and Methods

### 2.1. Samples and Cells

Iliac crest bone marrow aspirates were obtained from healthy donors at the Bone Marrow Transplant Unit, Hematology Service of the Clementino Fraga Filho University Hospital (HUCFF), at the Federal University of Rio de Janeiro, Rio de Janeiro, RJ, Brazil. All protocols and experimental procedures were approved by the Investigational Review Board at HUCFF. Mouse Wnt3a transfected L cells (L-Wnt3a) were obtained from the American Type Culture Collection (ATCC, Manassas, VA). Human breast cancer cell line MDA-MB-231 was obtained from the Rio de Janeiro Cell Bank (BCRJ, Rio de Janeiro, RJ, Brazil).

### 2.2. Antibodies and Reagents

The following primary antibodies were used: rabbit anti-Pan-cadherin (C3678, Sigma-Aldrich, St. Louis, MO) that recognizes the conserved C-terminal domain of classic cadherins, mouse anti-N-cadherin (clone 32) and anti-E-cadherin (clone 36), both from BD Biosciences (Franklin Lakes, New Jersey, USA), rabbit anti-*β*-catenin (Invitrogen-Molecular Probes, Carlsbad, CA), mouse anti-active-*β*-catenin (clone 8E7, Millipore, Billerica, MA, USA), mouse anti-lamin A∖C (BD Biosciences), and mouse anti-*α*-tubulin (clone DM1a, Sigma-Aldrich). Secondary antibodies were Alexa Fluor™ 488 goat anti-rabbit IgG, Alexa Fluor™ 546 rabbit anti-mouse IgG (Invitrogen, Life Technologies, Brazil, São Paulo, SP, Brazil), and peroxidase-conjugated goat anti-rabbit and rabbit anti-mouse (Promega, Madison, WI). DAPI dihydrochloride (Invitrogen) was used for nuclear staining. The *γ*-secretase activity inhibitor Dapt (N-N[-(3,5-Difluorophenacetyl-l-alanyl)]-S-phenylglycine-t-butyl-ester) was from Merck Biosciences (Darmstadt, Germany). Nuclear and cytoplasmic fractions were extracted using NE-PER® Nuclear and Cytoplasmic Extraction Reagents (Pierce Biotechnology, Rockford, IL).

### 2.3. Isolation and Culture of Human Bone Marrow Stromal Cells (BMSCs)

BMSCs were isolated as previously described [40]. Bone marrow collection kits were washed with phosphate buffered saline (PBS) after bone marrow aspirates were transferred to infusion bags. Cell suspensions were diluted 6 : 1 in Hespan® (hydroxyethyl starch saline, American Hospital Supply Corp., McGaw Park, IL) and incubated for 30 min at room temperature (RT) for hemosedimentation. Supernatants were collected, washed with PBS, plated at 1.0 × 10^6^ cells/mL in Dulbecco's medium (DMEM low-glucose, LGC, São Paulo, SP, Brazil) supplemented with 10% fetal bovine serum (FBS, Cultilab, Campinas, SP, Brazil) and antibiotics (100 U/mL of penicillin and 100 mg/mL of streptomycin, both from Sigma-Aldrich, St. Louis, USA), and incubated at 37°C in a humidified atmosphere containing 5% CO_2_. After 3 days, nonadherent cells were removed, and adherent cells were washed with PBS and maintained until 70% confluence. Cells were harvested by enzymatic digestion with 0.125% trypsin and 0.78 mM EDTA (both from Sigma-Aldrich) and expanded in DMEM with 10% FBS and antibiotics (expansion medium, EM).

### 2.4. Mouse L-Cell Culture and Wnt3a-Conditioned Medium Preparation

L cells were cultured in DMEM supplemented with 10% FBS and 0.4 mg/mL neomycin (Invitrogen) to maintain transgene expression during cell culture expansion. Conditioned medium from L-Wnt3a was collected according to the manufacturer's instructions and as described [41]. Briefly, 1.3 × 10^6^ cells were plated in 75 cm^2^ culture flasks with 14 mL of medium without antibiotics and left to grow for four days. The first batch of medium was collected and replaced with 14 mL of fresh medium for another three days. The second batch of medium was then collected and the cells discarded. Both batches were mixed, sterile-filtered (0.22 *μ*m), and stored at −20°C. The presence of Wnt3a protein in medium obtained with the same cell lineage used in this study has been shown previously in [42]. Activity of the conditioned medium was tested by the TCF/LEF luciferase reporter assay using MDA-MB-231 cells as described below and in previous studies of our group, using both HEK 293T cells and myoblasts [41, 42].

### 2.5. Osteogenic and Adipogenic Differentiation

BMSCs were plated at 2.5 × 10^4^ cells/cm^2^ and cultured in EM until confluence. Osteogenic differentiation was induced after the cells reached confluence. Cells were maintained for up to 21 days in osteogenic medium (OM), that is, DMEM containing 10% FBS and antibiotics, 5 *μ*g/mL ascorbic acid 2-phosphate, 10 mM *β*-glycerophosphate, and 10^−6^ M dexamethasone (all from Sigma-Aldrich). Dexamethasone at 10^−6^ M was shown to efficiently induce osteogenic differentiation of BMSC (Supplementary Figure 1 in Supplementary Material available online at http://dx.doi.org/10.1155/2016/3865315) and osteoblast cell lines [43] when the treatment is initiated after cells reach confluence. Medium was changed in every 2-3 days. Differentiation was evaluated by alkaline phosphatase activity and quantification of mineralized foci after Von Kossa staining. Adipogenic potential was evaluated by maintaining the cells in DMEM supplemented with 10% FBS, 0.5 mM isobutyl-methylxanthine (IBMX), 10^-6 ^M dexamethasone, 200 *μ*M indomethacin (all from Sigma-Aldrich), 10 *μ*M insulin (Humulin®, Lilly, São Paulo, SP, Brazil), and antibiotics for up to 21 days. Accumulation of cytoplasmic lipids was identified by Oil Red O staining.

### 2.6. PS1/*γ*-Secretase Activity Assay

To investigate the role of PS-1/*γ*-secretase in osteogenic differentiation of BMSCs, 20 *μ*M of Dapt [44] was added to the cultures 24 h prior to osteoinduction or 24 h, 48 h, and 4 days after addition of OM. Once added, Dapt was maintained throughout the differentiation protocol.

### 2.7. Wnt3a Treatment

Different concentrations (1%, 5%, or 10% v/v) of Wnt3a-conditioned medium (Wnt3a-CM) were added to EM or incomplete OM (iOM), which did not contain dexamethasone. During osteogenic induction, Wnt3a-CM was added to the cultures at the onset of osteoinduction (0 h) or 48 h and 4 days after control osteoinduction with OM. In all assays, medium was exchanged at every 3 days.

### 2.8. Alkaline Phosphatase Activity Assay

Alkaline phosphatase (ALP) activity was determined by colorimetric assay using an ALP kit (Labtest Diagnóstica, Lagoa Santa, MG, Brazil), following manufacturer instructions. BMSCs were osteoinduced under the different conditions stated above for 7 and 14 days and total protein extracts were obtained by scrapping the monolayers in 300 *μ*L of 125 mM Tris-HCl (pH 6.8) 0.5% Triton X-100 buffer. Equal volumes of substrate (22 mmol/L thymolphthalein monophosphate) and protein extracts were mixed and incubated in a water bath at 37°C for 10 min. Reaction was stopped by adding a 250 mmol/L NaOH 94 mmol/L Na_3_CO_2_ colorimetric solution. The optical density (OD) of the product was measured at 590 nm. Protein concentration of cell extracts was measured with Bradford Reagent (Sigma-Aldrich), and ALP activity was shown as (OD of test sample/OD of standard control sample) × 45/mg of total protein.

### 2.9. Von Kossa and Oil Red O Staining

BMSCs monolayers were fixed with 4% paraformaldehyde in PBS for 1 h at RT and stained with either Von Kossa or Oil red O as described [45]. Von Kossa staining was performed by incubating monolayers with 2% silver nitrate solution for 1 h in the absence of light. Monolayers were washed five times with water to remove excess stain and plates were then exposed to UV light for 10 minutes. For Oil Red O staining, monolayers were incubated for 2 minutes with propylene glycol and then with 0.5% Oil Red O in propylene glycol for 20 minutes. Monolayers were washed with 85% propylene glycol solution for 1 minute and finally twice with water. To quantify the extent of mineralization and the number of fat accumulating cells, wells were photographed using an inverted microscope (Nikon Eclipse TS100, Nikon, Tokyo, Japan) equipped with a EC3 digital camera (Leica, Wetzlar, Germany). The mineralized area in 15 random fields was quantified using NIH Image J software and is represented as the percentage of the total area. Number of fat accumulating cells is expressed as number of cells per field.

### 2.10. Proliferation Assay and Doubling Population

A total of 1.0 × 10^4^ BMSCs were plated per well in 24-well dishes in control expansion medium (EM) and left to adhere overnight. On the next day, cells from three sample wells were recovered with 0.125% trypsin 0.78 mM EDTA solution and counted in a Neubauer chamber. Viability was evaluated by the Trypan Blue exclusion method. The mean value obtained from these wells was considered as the initial number of adherent cells per well. The culture medium of the remaining wells was replaced by either EM or incomplete osteogenic medium (iOM) containing 2% FBS alone or supplemented with 1% or 10% Wnt3a conditioned medium. Cells in each well were harvested after 2, 6, 8, and 10 days of culture and quantified as described above. The number of population doublings was calculated using the following equation: PD = (_*f*_ *N* − _*i*_ *N*)/log_10_⁡(2), where _*f*_ *N* is the final cell harvest number and _*i*_ *N* is the initial cell number.

### 2.11. Clonogenic Assay (CFU-F, Colony-Forming Unit-Fibroblast)

CFU-F was performed by plating 100 cells/cm^2^ in quadruplicate with DMEM with 10% FBS [3]. Cultures were maintained for 14 days. After this period, cells were fixed with 4% paraformaldehyde and stained with 1% crystal violet. Colonies with more than 50 cells were counted.

### 2.12. Immunofluorescence and Confocal Microscopy

Immunofluorescence labeling for confocal microscopy (TCS SP5, Leica) was performed as described [46]. BMSCs were fixed with 4% paraformaldehyde in PBS for 10 min at RT, permeabilized with 0.5% Triton X-100 in PBS (PBS-T), and incubated with the primary antibodies rabbit anti-Pan-cadherin, mouse anti-N-cadherin, mouse anti-E-cadherin, or rabbit anti-*β*-catenin diluted 1 : 50 in PBS-T for 1 h at 37°C in a humid chamber. Subsequently, cells were incubated with the secondary antibodies for 1 h at 37°C in a humid chamber. Nuclei were stained with 0.1 *μ*g/mL DAPI in 0.9% NaCl. Control experiments with no primary antibodies showed only a faint background staining.

### 2.13. Protein Sample Preparations and Western Blotting

For total cell extract collection, BMSCs cultures were scrapped in Ripa buffer (0.05 M Tris-HCl pH 7.4; 0.15 M NaCl; 1% NP-40; 0.25% sodium deoxycholate, 2 mM EDTA) containing protease inhibitors (1 mg/mL aprotinin, 1 mg/mL leupeptin, 1 mg/mL pepstatin, 1 mM phenylmethylsulfonyl fluoride (PMSF), 10 mM N-ethylmaleimide, 1 mM sodium fluoride, and 1 mM sodium orthovanadate, all from Sigma-Aldrich). Nuclear and cytoplasmic fractions were extracted according to the manufacturer's instructions. All cell extracts were diluted 1 : 2 in SDS-PAGE buffer (125 mM Tris-HCl pH 6.8, 4% sodium dodecyl sulfate, 20% glycerol, 10%  *β*-mercaptoethanol, and 0.002% bromophenol blue) and boiled for 5 min. The amount of protein in each sample was determined with Bradford Reagent (Sigma-Aldrich), using bovine serum albumin as a standard. Protein electrophoresis and blotting were performed as described in [46]. Samples were loaded in 12% SDS-PAGE and transferred to polyvinylidenfluoride membranes (GE Healthcare Lifesciences, New Jersey, USA). Proteins immobilized on the membranes were blocked for 1 h at RT with 5% nonfat dry milk and incubated with the primary antibodies rabbit anti-Pan-cadherin, rabbit anti-*β*-catenin, mouse anti-active-*β*-catenin, mouse anti-lamin A∖C, or mouse anti-*α*-tubulin. Membranes were incubated with goat anti-rabbit or rabbit anti-mouse peroxidase-conjugated antibodies and the bands visualized using the Super Signal WestPico ECL Pierce kit (Pierce). Molecular weight of detected bands was estimated using the protein molecular weight standards Kaleidoscope (Bio-Rad, Hercules, CA, USA) and Rainbow (GE Healthcare Lifesciences). Densitometric analysis was performed in scanned images (Scanjet G2710, HP, CA, USA) using Image J software.

### 2.14. Cell Electroporation, Lentivirus Transduction, and Luciferase Assay

Electroporation was performed as described [47]. A total of 5.0 × 10^5^ BMSCs were resuspended in 100 *μ*L electroporation buffer (5 mM KCl; 15 mM MgCl2; 120 mM Na_2_HPO_4_ pH7.2; 25 mM Sodium Succinate; 25 mM Manitol) containing 4 *μ*g of the reporter system 7xTcf-FFluc//SV40-PuroR (7TFP, Addgene plasmid 24308) to evaluate the activation of Wnt signaling [48] and 0.4 *μ*g of TK-Renilla (Promega). The cells were immediately transferred to a sterile 0.2 cm cuvette (Mirus Biotech®, Madison, WI, USA) and electroporated using the program U23 of the Lonza® Nucleofactor® II electroporation system. After transfection, 1.0 × 10^5^ cells were plated per well in 48-well plates and left to adhere overnight at 37°C and 5% CO_2_. On the following day, cultures were rinsed with PBS and incubated in triplicate with EM, OM, OM supplemented with 20 *μ*m Dapt, iOM, or iOM supplemented with 10% and 50% Wnt3a-CM for 48 h or 5 days. As internal controls, MDA-MB-231-7TFP reporter lineages were also obtained by either electroporation, using the same conditions described above, or by lentivirus transduction [49]. Cells were incubated overnight with lentiviral particles containing the 7TFP sequence in the presence of 8 *μ*g/mL polybrene in Iscove's Modified Medium (IMDM) containing 10% FBS. After incubation, the medium was replaced and 2 *μ*g/mL puromycin (Invitrogen, A1113802) was added to select the transduced cells. After the specific treatments, cells were lysed with lysis buffer (Promega) and luciferase activity was detected by adding the enzyme substrate according to the manufacturer's protocol. Samples were read in a microplate reader (Modulus II, Turner Biosystems, CA, USA). To normalize the data, the luciferase activity index was calculated by dividing the luciferase values by the Renilla luciferase values.

### 2.15. Real-Time Polymerase Chain Reaction (RT-PCR)

mRNA from undifferentiated, 48 h and 5 days osteoinduced cells were isolated using Trizol (Invitrogen) reagent, according to the manufacturer's instructions, and quantified using a Nanodrop spectrophotometer. Two micrograms of total RNA was used as a template for cDNA synthesis, using the High Capacity cDNA Reverse Transcription kit (Life Technologies). SYBR Green PCR master mix (Life Technologies) was used to quantify human Axin-2 and Hes-1 expression levels, with GAPDH as an endogenous control. Real-time reactions were performed in triplicate using a Line Gene 9600 Real-Time thermocycler (Bioer). Relative quantification was performed using the Delta-Delta Ct method. Primer sequences were as follows: Axin-2, forward: 5′-GTCTCTACCTCATTTCCCGAGAAC-3′, reverse: 5′-CGAGATCAGCTCAGCTGCAA-3′; Hes-1: forward: 5′-AGAAAGATAGCTCGCGGCATT-3′, reverse: 5′-GGTGCTTCACTGTCATTTCCA-3; GAPDH, forward: 5′-ACTGTGTTGGCGTACAGGTC-3′, reverse: 5′-CATGAGTCCTTCCACGATACCA-3′.

### 2.16. Statistical Analysis

Statistical analysis was carried out using the GraphPad Prism software version 5. Results of at least three independent experiments (always performed with cells isolated from different donors) were compared by One-Way ANOVA. Differences between groups were evaluated with the posttest of Tukey. Data are shown as mean ± standard deviation (SD). *p* values < 0.05 were considered significant.

## 3. Results

### 3.1. *β*-Catenin/TCF Signaling Is Activated during Osteogenic Differentiation of BMSCs But Is Not Induced by Wnt3a-CM

**Figure 1:**
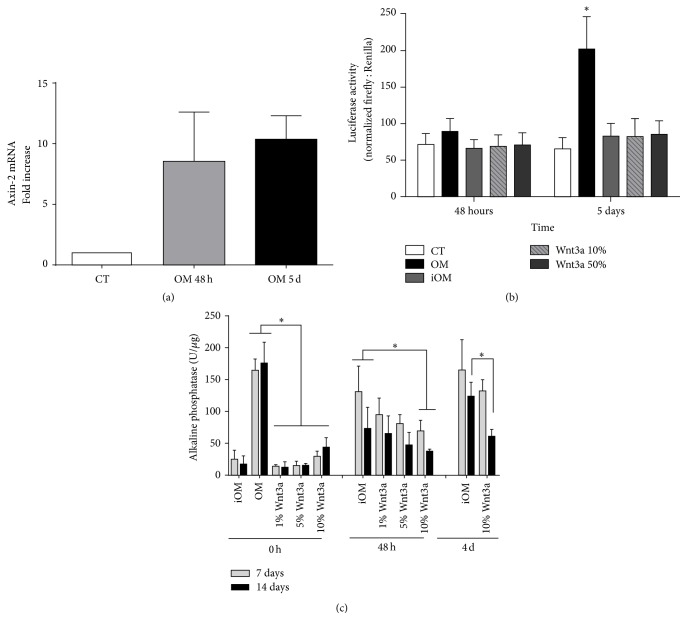
Osteogenic medium, but not Wnt3a-CM, induces *β*-catenin signaling and BMSCs osteogenic differentiation. (a) BMSCs were cultured in osteogenic medium (OM) for 48 h and 5 days and Axin-2 mRNA fold increase was evaluated. Bars represent mean ± SD of 3 independent experiments performed in triplicate. (b) Luciferase activity in BMSCs containing a SuperTop TCF reporter, osteoinduced for 48 h and 5 days with incomplete osteogenic medium (iOM, without dexamethasone), osteogenic medium (OM), or iOM containing 10% or 50% Wnt3a-CM. CT = undifferentiated cells. Bars represent mean ± SD of 4 experiments performed in triplicate. (c) Alkaline phosphatase activity in BMSC cultures after 7 and 14 days of osteoinduction with OM or iOM containing 1%, 5%, and 10% Wnt3a-CM. iOM supplemented with Wnt3a-CM was added at the onset of induction (0 h) or after 48 h and 4 days of osteoinduction with OM. Mean ± SD of 5 independent experiments with triplicates is shown. ^*∗*^ *p* < 0.05.

To confirm the activation of *β*-catenin/TCF signaling during BMSCs osteoinduction, we first evaluated the expression of Axin-2, a known *β*-catenin/TCF target [50]. After 48 h of BMSCs incubation with osteogenic medium containing dexamethasone (OM), Axin-2 mRNA levels were upregulated; and this increased expression was maintained even after 5 days of osteoinduction (Figure 1(a)). To confirm this observation, we transfected BMSCs with a plasmid containing a luciferase reporter gene, downstream of seven *β*-catenin/TCF binding sites. A significant luciferase activity was observed in cells treated for 5 days with OM (Figure 1(b)), thus confirming *β*-catenin signaling activation upon osteoinduction. Next, we asked whether canonical Wnt3a would mimic this effect and induce *β*-catenin signaling in BMSCs. We observed that the replacement of dexamethasone by 10% or 50% Wnt3a-conditioned medium (Wnt3a-CM) did not increase luciferase activity in BMSCs over basal levels (Figure 1(b)). As parallel experiments performed with MDA-MB-231 cells, as internal controls, showed a significant luciferase induction upon Wnt3a-CM treatment (Supplementary Figure 2), we thereby concluded that Wnt3a was unable to induce *β*-catenin signaling in BMSCs.

To further investigate the effects of Wnt3a treatment in BMSCs osteoinduction, we then evaluated alkaline phosphatase (ALP) activity, an enzyme upregulated in the first week of the* in vitro* osteogenic program [51]. After 7 and 14 days of BMSCs treatment with 1%, 5%, or 10% Wnt3a-CM, no increases in ALP were observed in any concentrations of Wnt3a-CM tested, as opposed to cells induced with OM (Figure 1(c)). Considering this finding, we next asked whether Wnt3a was only insufficient to trigger the osteogenic program or was actually inhibiting differentiation. To test this hypothesis, we preosteoinduced BMSCs with OM for 48 hours or 4 days and then replaced dexamethasone by 1%, 5%, or 10% Wnt3a-CM. In the 48 h preosteoinduced cells, we observed a dose-dependent decrease in OM-induced ALP activity, which became significant (*p* < 0.05) at the concentration of 10% Wnt3a-CM (Figure 1(c)). Similar results were observed in the cells preosteoinduced for 4 days (Figure 1(c)), supporting the notion that Wnt3a can inhibit an ongoing osteogenic process.

**Figure 2:**
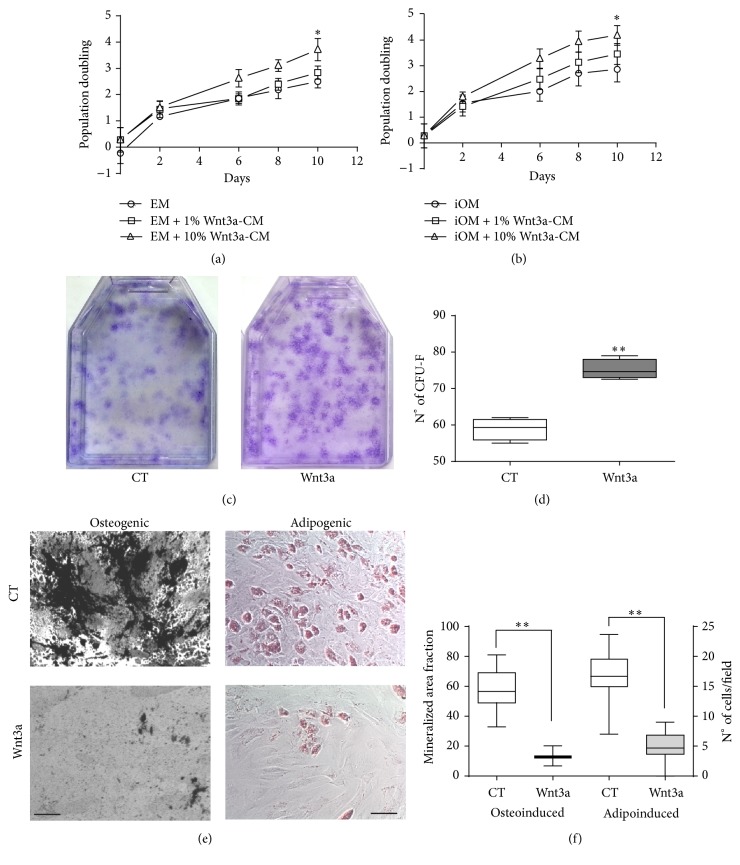
Wnt3a-CM stimulates proliferation and impairs BMSCs differentiation. (a-b) Cumulative population doublings of BMSCs cultured with either expansion medium (EM (a)) or incomplete osteogenic medium (iOM, without dexamethasone (b)) supplemented with 1% or 10% Wnt3a-CM. Data represent mean ± SD of 3 independent experiments with triplicates. (c-d) BMSCs expanded in the presence of 10% Wnt3a-CM for 10 days were plated at clonal density and maintained in control expansion medium for another 14 days. Morphology (c) and quantification (d) of CFU-F formed by control (CT) and Wnt3a-expanded BMSCs are shown. Data represent mean ± SD of 4 experiments with quadruplicates. (e) Representative micrographs of control and Wnt3a-expanded BMSCs induced towards the osteogenic (left panel) and adipogenic lineages (right panel) stained with Von Kossa and Oil Red O, respectively. Bars = 100 *μ*m. (f) Quantification of total mineralized area and fat accumulating cells per field of view. Data represent mean ± SD of 4 experiments with triplicates. ^*∗*^ *p* < 0.05; ^*∗∗*^ *p* < 0.001.

In fact, we noticed that Wnt3a treatment seemed to stimulate and maintain a proliferative cellular state. To confirm this observation, we cultured BMSCs with either expansion medium (EM) or incomplete OM (iOM, without dexamethasone), each containing 1% or 10% Wnt3a-CM. After 10 days, cells expanded in the presence of 10% Wnt3a-CM had an increased number of population doublings, indicative of a higher proliferative rate (Figures 2(a)-2(b)). Moreover, when replated in clonal density, a higher number of colonies originated from Wnt3a-CM expanded cells (Figures 2(c)-2(d)), pointing to an enhancement in clonogenic potential (1 colony/33.23 cells compared to 1 colony/42.43 in control cells). Lastly, when subjected to standard* in vitro* differentiation, Wnt3a-expanded cells had a decreased capacity for both matrix mineralization and lipid accumulation (Figures 2(e)-2(f)). Therefore, we concluded that Wnt3a-mediated signaling induces a proliferative status in BMSCs, impairing differentiation programs.

### 3.2. Cadherins Are Cleaved by a PS1/*γ*-Secretase-Mediated Mechanism during BMSCs Osteoinduction

**Figure 3:**
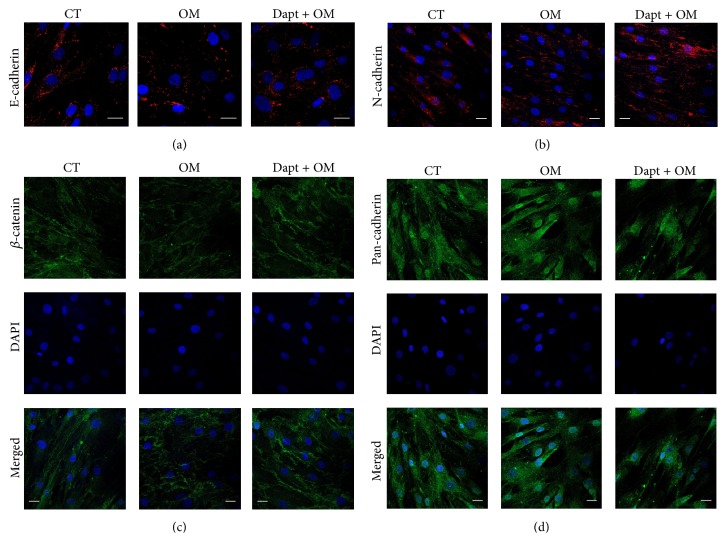
Expression of E-cadherin, N-cadherin, and *β*-catenin in osteoinduced and Dapt-treated BMSCs. Confocal microscopy images showing the expression of E-cadherin ((a) red), N-cadherin ((b) red), *β*-catenin ((c) green), and Pan-cadherin ((d) green) by BMSCs cultured for 48 h with expansion medium (CT, control undifferentiated), osteogenic medium (OM), or osteogenic medium containing 20 *μ*M of the PS1/*γ*-secretase inhibitor Dapt (Dapt + OM). Dapt was added 24 h before the addition of OM. Nuclei were stained with DAPI (blue). Scale bars = 25 *μ*m.

Next we investigated the occurrence of cadherin cleavage in BMSCs. We first verified that both undifferentiated and 48 h-osteoinduced BMSCs expressed E-cadherin (Figure 3(a)) and N-cadherin (Figure 3(b)), in a linear/punctate pattern at the plasma membrane, and punctate in the cytosol. A similar membrane staining pattern was observed for *β*-catenin (Figure 3(c)). However, when 20 *μ*M of Dapt—a PS1 specific inhibitor—was added to OM, a stronger and more defined membrane staining of both N-cadherin (Figure 3(b)) and *β*-catenin (Figure 3(c)) was observed, suggesting a reduced turnover of these proteins at the plasma membrane.

**Figure 4:**
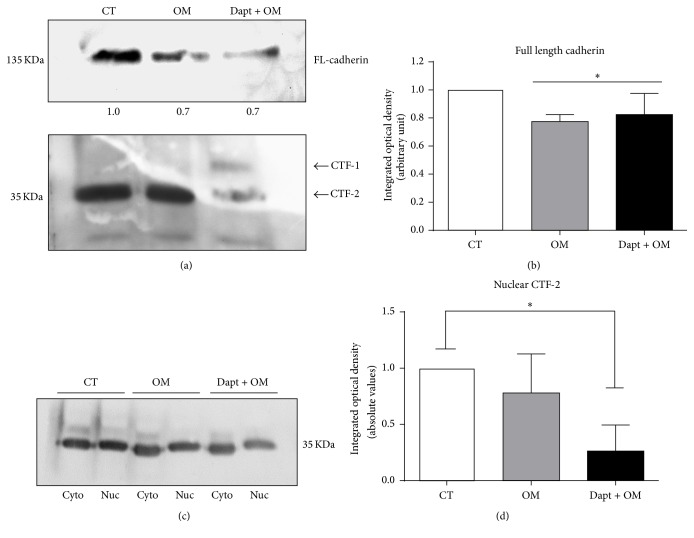
PS1/*γ*-secretase-mediated cadherin cleavage occurs during osteogenic differentiation of BMSCs, generating a C-terminal intracellular fragment (CTF-2) that translocates to nucleus. BMSCs were osteoinduced for 48 h in the presence of Dapt. (a) Representative immunoblotting showing a full-length cadherin band (135 kDa) and its cleavage products (CTF-1 and CTF-2) in total protein extracts. (b) Densitometric quantification of the 135 kDa cadherin band. Bars show mean ± SD of 4 independent experiments. (c) Representative immunoblotting showing CTF-2 fragments (35 kDa) in cytoplasmic and nuclear fractions. (d) Quantification of nuclear CTF-2 in 3 experiments. CT = control undifferentiated cells; OM = osteogenic medium; Dapt + OM = osteogenic medium with Dapt. ^*∗*^ *p* < 0.05 relative to CT.

Because both E-cadherin and N-cadherin are targets of the PS1/*γ*-secretase complex [29, 30, 35, 52, 53], we investigated the occurrence of cadherin cleavage in BMSCs with a Pan-cadherin antibody that specifically recognizes the conserved C-terminal region of classic cadherins [54]. With this approach, a similar staining pattern of cadherins as of the specific previous antibodies was observed (Figure 3(d)). However, we also observed a staining in the nucleus (Figure 3(d)), which strengthened the hypothesis that cadherins were cleaved and its CTF-2 were translocated to the nucleus. To confirm this finding, we analyzed whole protein extracts of undifferentiated and 48 h-osteoinduced BMSCs by western blot, using the Pan-cadherin antibody. We observed a 135 kDa band, accompanied by a 35 kDa fragment (Figure 4(a)), which, respectively, agrees with the molecular weights of N-cadherin and its CTF-2 [29, 30]. However, the 135 KDa band was significantly decreased in osteoinduced cells (Figures 4(a)-4(b)). In Dapt-treated cells, an additional 40 kDa band was detected (Figure 4(a)), in agreement with the expected molecular weight of CTF-1, which is only detected when PS1/*γ*-secretase activity is inhibited [29, 30]. We also observed a decrease in the 35 kDa band (Figure 4(a)), corroborating the notion that the latter is originated from the former, after PS1/*γ*-secretase cleavage. We then evaluated the presence of the 35 kDa band (CTF-2) in isolated cytosolic and nuclear fractions, since solubilized CTF-2 might translocate to the nucleus [37]. As expected, CTF-2 was detected in both cellular compartments (Figure 4(c)), but its nuclear expression was diminished in Dapt-treated cells (Figure 4(d)). Taken collectively, these results indicate that cadherins are cleaved during osteogenic differentiation of BMSCs by a PS1/*γ*-secretase-dependent mechanism, generating soluble fragments that are translocated to the nucleus.

### 3.3. Pharmacological Inhibition of PS1/*γ*-Secretase during Osteoinduction Reduces *β*-Catenin Nuclear Translocation and Signaling

**Figure 5:**
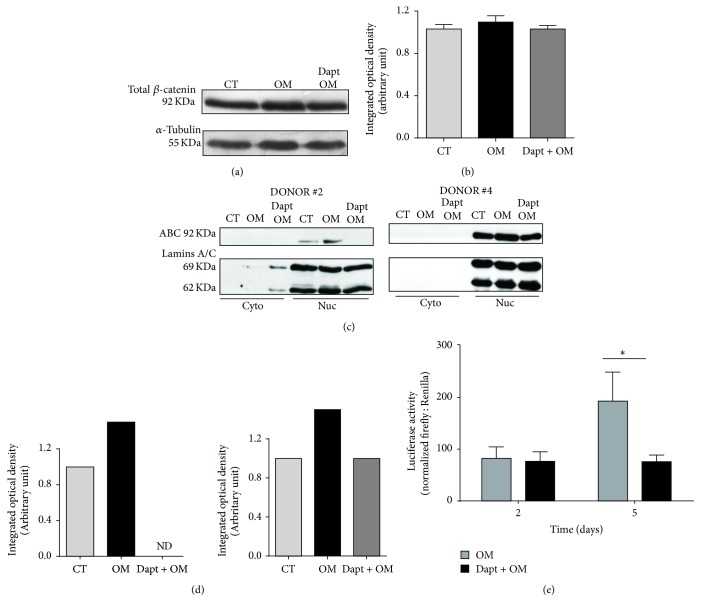
PS1/*γ*-secretase inhibition impairs *β*-catenin nuclear translocation and signaling. (a) Representative immunoblotting of total *β*-catenin in whole protein extracts. (b) Densitometry of total *β*-catenin bands obtained in 5 independent experiments. Data represent mean ± SD. (c) Representative immnunoblotting of the active form of *β*-catenin (ABC) in cytoplasmic and nuclear cell fractions from two different donors. (d) Densitometry of ABC nuclear bands normalized by lamin A. (e) Luciferase activity in BMSCs containing a SuperTop TCF reporter, osteoinduced for 2 and 5 days in the presence of Dapt. Bars represent mean ± SD of 5 different donors performed in triplicate. CT = control undifferentiated cells; OM = osteogenic medium; Dapt + OM = osteogenic medium with Dapt. ^*∗*^ *p* < 0.05.

We then sought to investigate the dynamics of *β*-catenin expression and signaling under PS1/*γ*-secretase inhibition. Analysis of the total amount of *β*-catenin showed no significant differences in expression after 48 hours of induction (Figures 5(a) and 5(b)). However, using an antibody that specifically recognizes the active (unphosphorylated) signaling form of *β*-catenin (ABC) [55, 56], we detected an increase in its nuclear localization at 48 h of osteoinduction (Figures 5(c) and 5(d)). On the other hand, Dapt-treated cells showed a reduced accumulation of ABC in the nucleus (Figures 5(c) and 5(d)). This effect was observed in cells isolated from different donors, though in different intensities of response. To confirm this observation, we evaluated the levels of luciferase activity in BMSCs transfected with the TCF reporter system and observed a significant impairment of *β*-catenin/TCF signaling after 5 days of osteoinduction in the presence of Dapt (Figure 5(e)). Taken altogether, the data indicate that *β*-catenin nuclear translocation and signaling during BMSCs osteoinduction depend on PS1/*γ*-secretase activity.

### 3.4. Osteogenic Differentiation Is Impaired under PS1/*γ*-Secretase Inhibition

**Figure 6:**
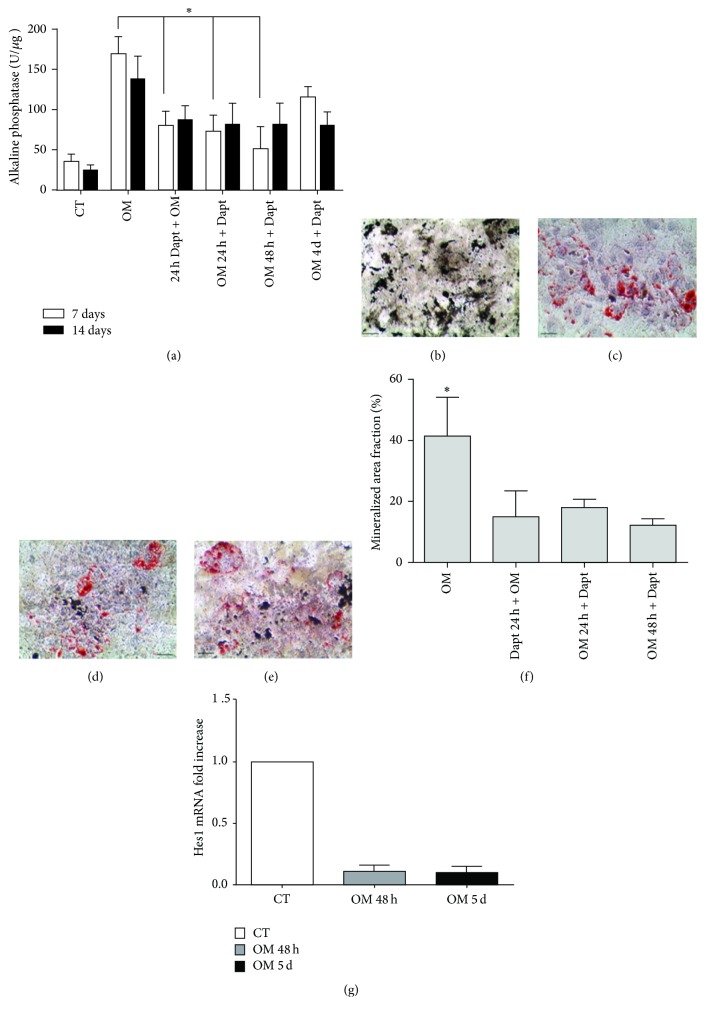
PS1/*γ*-secretase inhibition impairs BMSCs osteogenic differentiation. BMSCs were osteoinduced in the presence of Dapt, added either 24 hours before OM (24 h Dapt + OM) or at 24 h (OM 24 h + Dapt), 48 h (OM 48 h + Dapt), or 4 days after OM (OM 4 days + Dapt). (a) Alkaline phosphatase activity measured after 7 and 14 days. Graph represents the mean ± SD of 6 independent experiments. (b–e) Von Kossa and Oil Red O double staining. (b) Control osteoinduced, (c) 24 h Dapt + OM, (d) OM 24 h + Dapt, and (e) OM 48 h + Dapt. Scale bars = 100 *μ*m. Data are representative of 4 independent experiments. (f) Quantification of total mineralized area revealed by Von Kossa staining. Data represent mean ± SD of 3 independent experiments. ^*∗*^ *p* < 0.05. (g) Analysis of Hes-1 mRNA in BMSCs osteoinduced for 48 h and 5 days. Bars represent the mean ± SD of 3 independent experiments performed in triplicate.

To verify whether PS1/*γ*-secretase inhibition would indeed impact the acquisition of the osteoblastic phenotype, Dapt was added to BMSCs at different time points, starting 24 h before osteoinduction, or later at 24 h, 48 h, or 4 days after induction with OM. We verified that Dapt inhibited the induction of ALP activity, but only when treatment commenced before osteoinduction or during its first 48 h (Figure 6(a)). In these conditions, the mineralized area fraction was significantly reduced at day 21 (Figures 6(b)–6(f)), and fat accumulating cells appeared (Figures 6(c)–6(e)), indicating a shift from the osteogenic to the adipogenic program.

Although Notch signaling is known to be a negative regulator of osteogenic differentiation and shown to be inhibited upon osteogenic commitment [57–60], we lastly evaluated Notch regulation in order to verify its possible interplay in our observations. Analysis of Hes-1, the intracellular mediator of Notch [59], confirmed that this gene is significantly downmodulated in both 48 h- and 5 day-osteoinduced cells (Figure 6(g)). Considering that Dapt treatment would further inhibit Notch and therefore enhance osteogenic differentiation, we concluded that this signaling pathway does not take part in the results observed hereby.

## 4. Discussion

Bone marrow stromal cells (BMSCs) have a high potential for bone bioengineering and clinical application [5, 6]. However, the molecular mechanisms that drive commitment and differentiation along the osteoblastic lineage are not completely understood. Because evidence showed that receptor-mediated canonical Wnt signaling would be just one of the players of a major and intricate signaling complex responsible for cell fate decision, in which cadherin molecules also participate [19, 20, 24], we focused on understanding how these signaling pathways influence downstream *β*-catenin signaling and osteogenic differentiation. We observed that Wnt3 was not able to stimulate *β*-catenin signaling, maintaining BMSCs in a proliferative state. On the other hand, PS1/*γ*-secretase activity occurs upon osteoinduction, cleaving N-cadherin and enhancing *β*-catenin signaling. The inhibition of PS1/*γ*-secretase activity was associated to the impairment of osteogenic differentiation.

The specific effects of Wnt signaling activation in BMSCs were explored in several studies with conflicting results, either stimulating or inhibiting osteogenic differentiation [16–18, 61, 62]. More recently, Caverzasio and colleagues further explored the issue and strengthened the notion that Wnt3a stimulates BMSC proliferation by a *β*-catenin-independent pathway [61]. This kind of signaling transactivation mediated by canonical Wnts has been previously observed in other systems, such as during morphogenetic movements in vertebrate gastrulation [63], and is thought to be evolutionarily ancient [14]. Since we did not observe *β*-catenin/TCF activation in Wnt3a-treated cells, we hypothesize that the enhanced proliferation observed is probably related to the activation of an alternative, *β*-catenin-independent pathway.

However, we did observe that cells osteoinduced under standard conditions (with the osteogenic cocktail) showed an increased expression of the *β*-catenin responsive gene Axin-2, as well as an approximately 4-fold increase in TCF activation, confirming that *β*-catenin signaling is activated during the osteogenic program. Therefore, considering that (i) *β*-catenin is a component of cadherin adhesion complexes [64, 65]; (ii) stable expression of N-cadherin in cells of the osteoblastic lineage inhibits differentiation and impairs bone formation [66]; (iii) N-cadherin expression is downmodulated upon osteogenic commitment and differentiation [21, 23, 26–28]; and (iv) cadherin cleavage can influence *β*-catenin availability for signaling [31, 33, 36–38, 52, 53], we investigated whether this mechanism would influence *β*-catenin/TCF signaling activation during BMSCs osteoinduction. To date, this mechanism had not been shown during osteogenic differentiation of human BMSCs.

Consistent with our hypothesis, western blotting analysis showed a significant expression of a 135 kDa cadherin molecule, which was decreased after osteoinduction, even in the presence of Dapt. This observation was not surprising, once the first step of cleavage is MMP-dependent [31, 38]. Most importantly, as seen in previous reports [29, 30, 34], a 35 kDa cadherin fragment was observed in both undifferentiated and osteoinduced cells; and when Dapt was added, its expression was diminished and an additional 40 kDa fragment was observed, indicating that these fragments were, respectively, the cleavage product and the substrate of PS1/*γ*-secretase. The molecular weight of the full-length protein and its fragments suggests that N-cadherin [29, 30, 34] is the major target of PS1/*γ*-secretase in BMSCs. However, it must be considered that the amount of unprocessed E-cadherin might be under the limit of detection of the assay, and therefore, we cannot rule out that E-cadherin is also cleaved.

In several cell lineages, cadherin cleavage by PS1/*γ*-secretase released *β*-catenin from cell-adhesion complexes, favoring its translocation to the nucleus [30, 31, 33, 36–38, 52, 53]. Here we observed that, following osteoinduction, the amount of active, nonphosphorylated *β*-catenin (ABC) increased in the nucleus and resulted in TCF activation. However, this effect was impaired in all Dapt-treated cells, isolated from different donors. It is important to note that BMSCs are a highly heterogeneous population, containing progenitors in different levels of commitment [1, 67–69]. Therefore, the differences seen in the intensity of *β*-catenin translocation impairment among the different samples are not unexpected but are rather an intrinsic characteristic of the population. Furthermore, the pharmacological inhibition of PS1/*γ*-secretase significantly reduced TCF activation in the luciferase assays—which also varied among donors (23.6% to 69.8% in range)—confirming that *β*-catenin-mediated gene transcription in BMSCs is dependent on PS1/*γ*-secretase activity.

In agreement with the observed impairment of *β*-catenin signaling by PS1/*γ*-secretase inhibition, we found that the differentiation of BMSCs down to the osteoblastic lineage was arrested and redirected towards the alternative adipogenic program. This adipogenic enhancement following BMSCs treatment with Dapt was also described previously by Vujovic and colleagues [44], in a dose-dependent manner.

Although we were not able to confirm our observations with cells expressing cleavage-resistant cadherin molecules, which was a limitation of this study, our data demonstrate for the first time that BMSCs osteogenic differentiation is dependent on PS1/*γ*-secretase activity, which positively regulates both cadherin cleavage and *β*-catenin/TCF signaling, suggesting that this might indeed constitute a signaling axis leading to osteogenic commitment. This hypothesis is also strengthened by the observation that the expression of Hes-1 is inhibited upon BMSCs osteoinduction, which weakens the possibility of Notch signaling involvement in our findings. In this way, our study adds to the current literature as it provides evidence that both canonical Wnt and cadherin cleavage are central mechanisms of a complex and integrated net of signaling pathways responsible for BMSCs fate decisions, but with distinct outcomes. A better understanding of the specific roles of each mechanism in BMSCs, as well as when and how to modulate their function, will be a fundamental step for the development of effective strategies to improve BMSCs-based bone bioengineering and repair.

## 5. Conclusions

Osteogenic differentiation of BMSCs depends on the activity of PS1/*γ*-secretase, which cleaves cadherins and stimulates *β*-catenin signaling. In contrast, Wnt3a signaling is related to the maintenance of a proliferative state. Altogether, we provide evidence that corroborate the notion that cadherins and receptor-mediated Wnt signaling are central players in a major signaling pathway that can be differentially balanced leading to either BMSCs proliferation or differentiation.

## Supplementary Material

In supplementary figure 1, we show that, once BMSCs cultures are confluent, a higher concentration of dexamethasone is more efficient in inducing osteogenic differentiation. Therefore, this concentration was used in our experiments instead of the standard concentration used in the literature. In supplementary figure 2, we used the MDA-MB-231 humand breast cancer cell lineage as a positive control to evaluate the activity of the Wnt3a-conditioned medium used in our experiments.
