# Supplementary figures and images for: PS1/γ-Secretase-Mediated Cadherin Cleavage Induces β-Catenin Nuclear Translocation and Osteogenic Differentiation of Human Bone Marrow Stromal Cells
Source: Stem Cells Int. 2016 Dec 8;2016:3865315. doi: 10.1155/2016/3865315 (PMC5178376; doi:10.1155/2016/3865315)

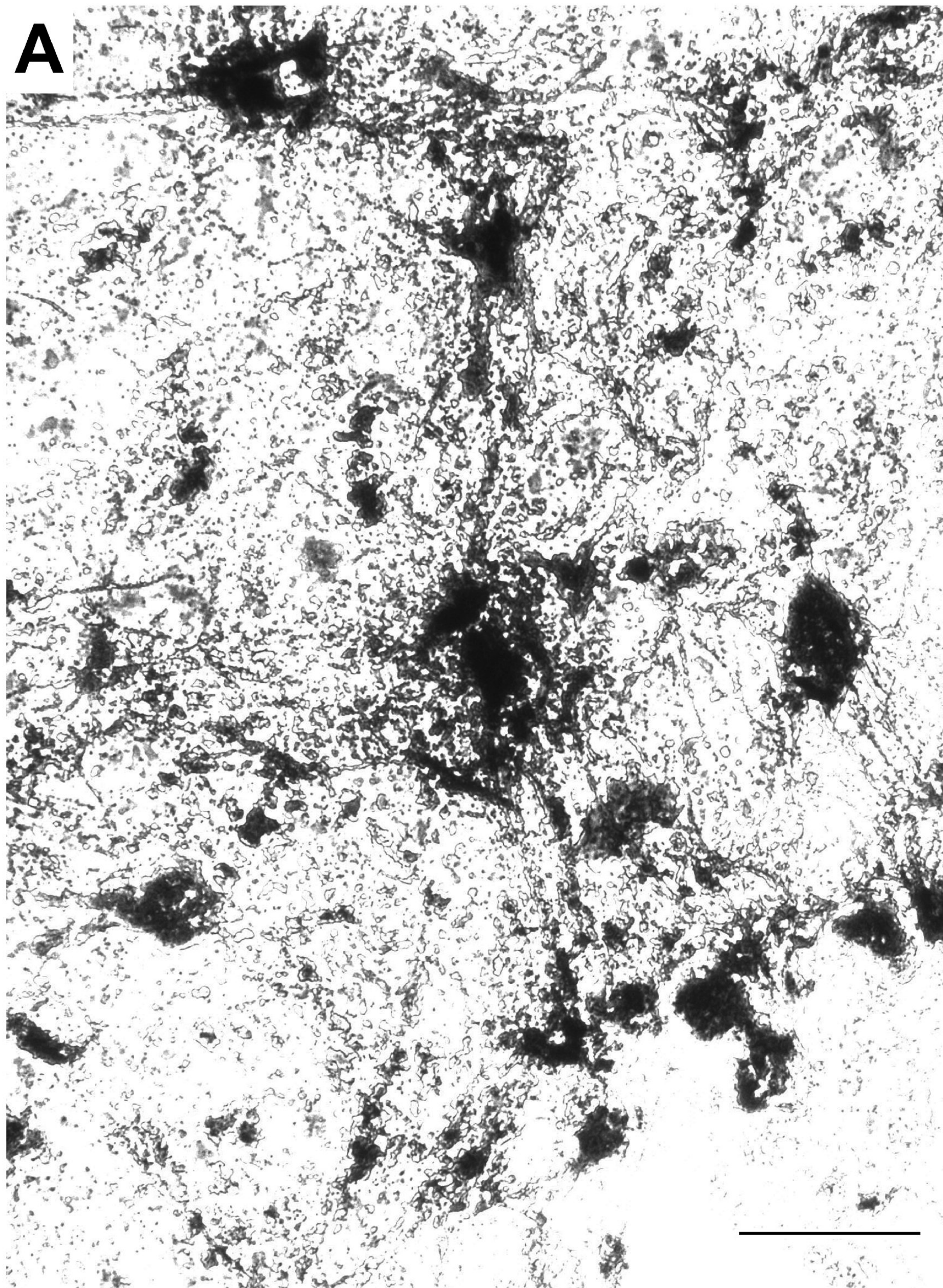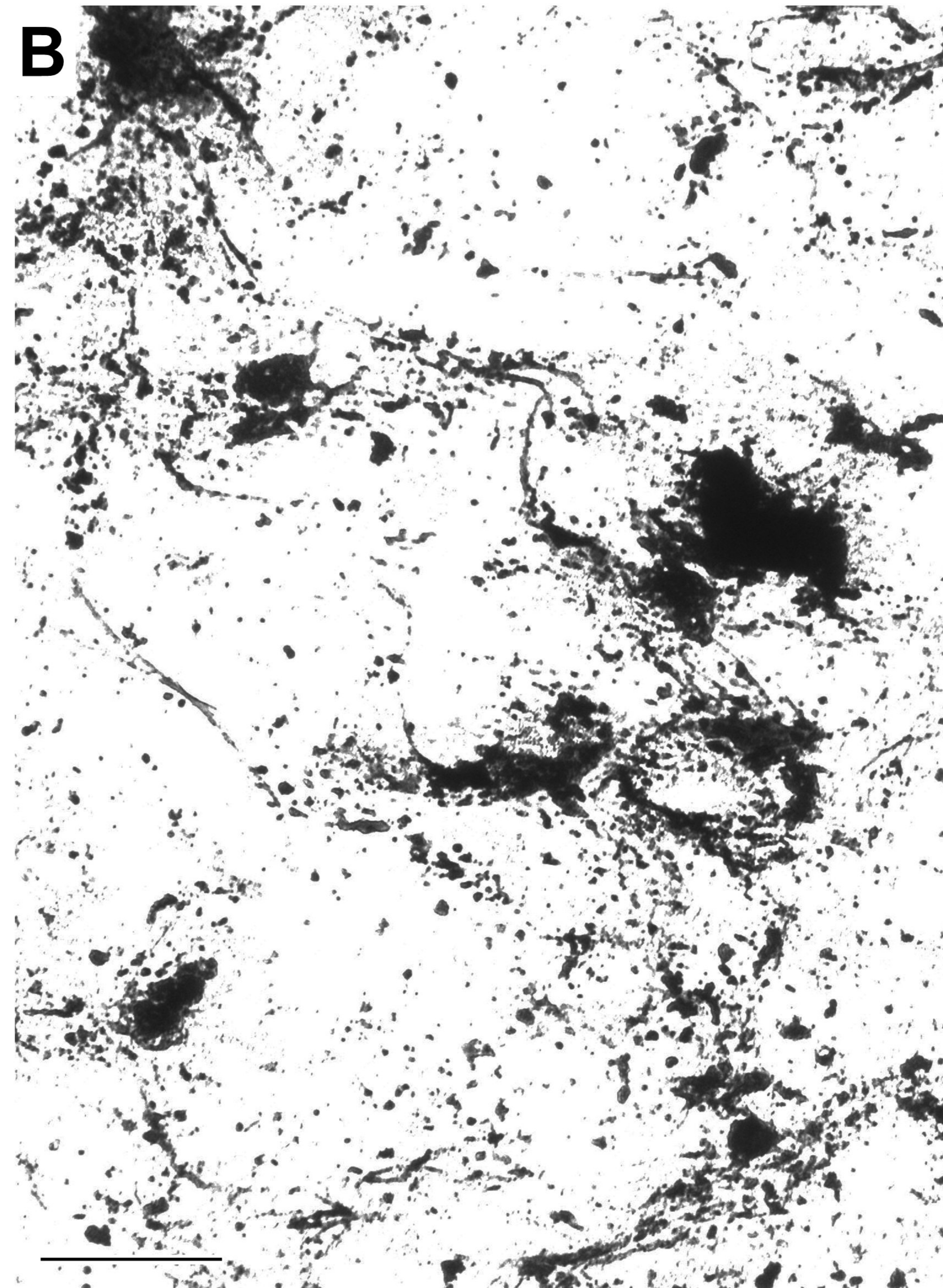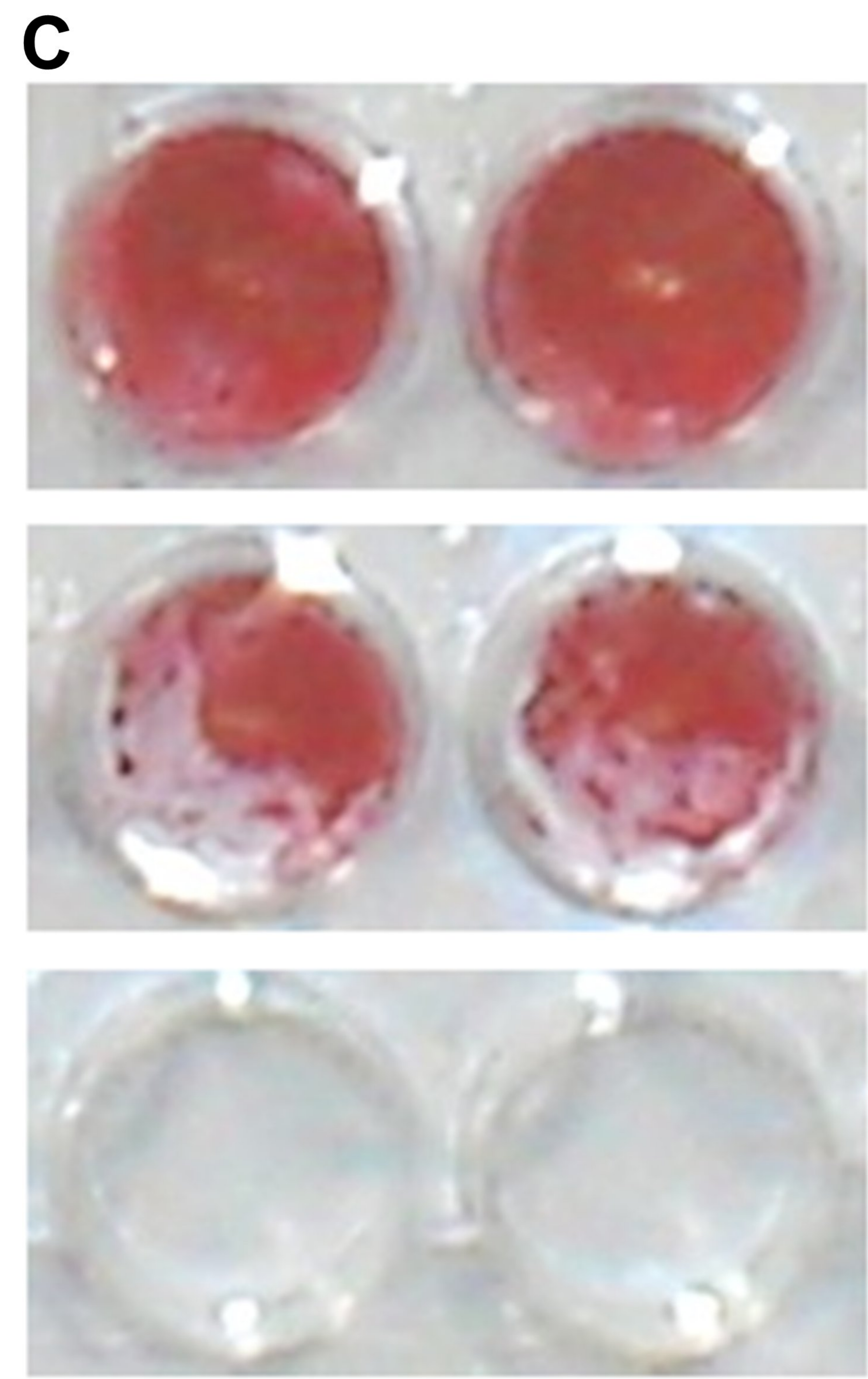

**Dex  $10^{-6}$ M**

**Dex  $10^{-8}$ M**

**CT**

Supplement: Supplementary file 1 — In supplementary figure 1, we show that, once BMSCs cultures are confluent, a higher concentration of dexamethasone is more efficient in inducing osteogenic differentiation. Therefore, this concentration was used in our experiments instead of the standard concentration used in the literature. In supplementary figure 2, we used the MDA-MB-231 humand breast cancer cell lineage as a positive control to evaluate the activity of the Wnt3a-conditioned medium used in our experiments. [file 3865315.f1.pdf]

**A**

MDA-MB-231

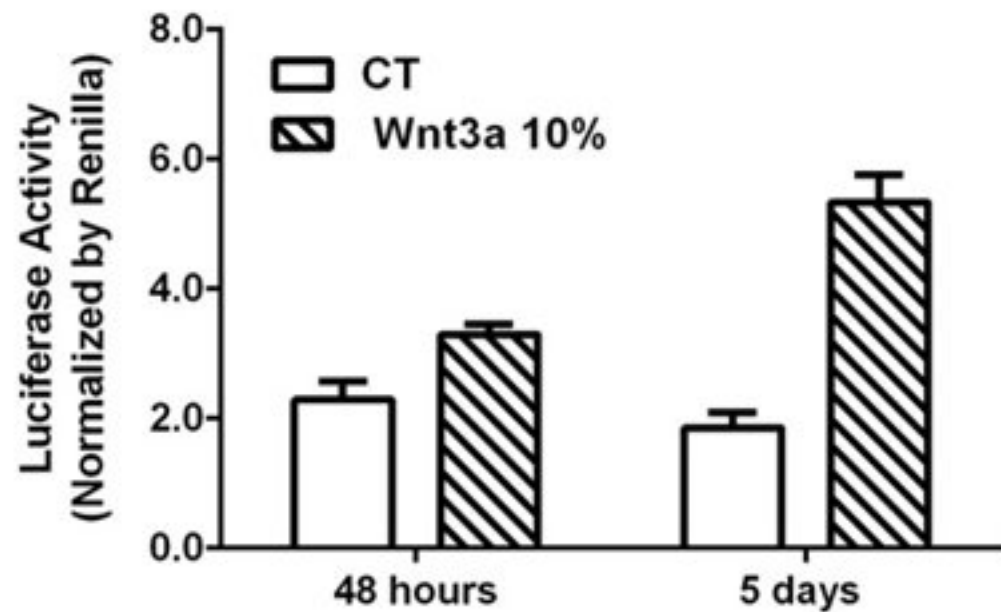**B**

MDA-MB-231

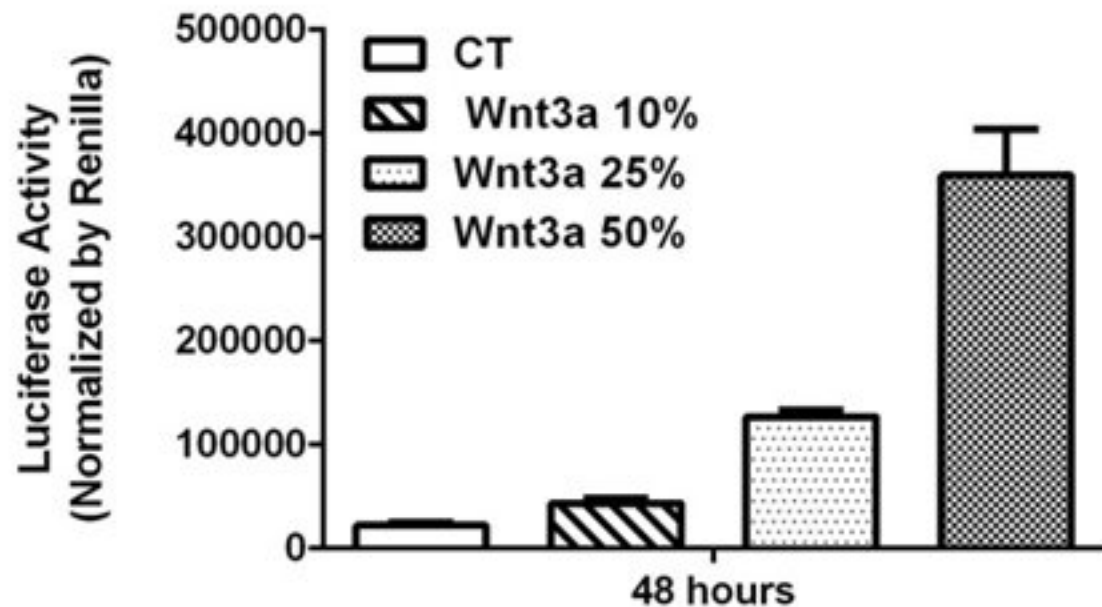

Supplement: Supplementary file 2 [file 3865315.f2.pdf]
